# Lateral neck cyst surgery without ipsilateral tonsillectomy: a retrospective analysis

**DOI:** 10.1007/s00405-022-07542-0

**Published:** 2022-07-19

**Authors:** Franziska E. Schwan, Julian Künzel, Florian Weber, Veronika Vielsmeier, Christopher Bohr, Kornelia E. C. Andorfer

**Affiliations:** 1 Department of Otorhinolaryngology, University Hospital Regensburg, Franz-Josef-Strauss-Allee 11, 93053 Regensburg, Germany; 2 Department of Pathology, University Hospital Regensburg, Franz-Josef-Strauss-Allee 11, 93053 Regensburg, Germany

**Keywords:** Branchiogenic cyst, Lateral neck cyst, Lymphoepithelial cyst, Tonsillectomy

## Abstract

**Purpose:**

Several theories have been proposed regarding the origin of lateral neck cysts (LNC). Besides complete surgical resection ipsilateral tonsillectomy and dissection of a tract or its remnants is sometimes recommended. In this retrospective trial we wanted to evaluate if patients, who received LNC resection only, develop complications or recurrence to justify this surgical strategy.

**Methods:**

Patients who received LNC resection between 2004 and 2017 at the Ear Nose and Throat Department of a university hospital were included. Data was collected from the clinic database and through a structured telephone interview.

**Results:**

A total of 126 patients met the inclusion criteria. In this collective, the diagnosis of a lateral neck cyst was confirmed histologically. Mean age at time of operation was 38 years (± 14.6). The median follow-up time was 7 years (range 3–18). None of the participants experienced recurrent unilateral pharyngitis or tonsillitis during follow-up. Furthermore, there was no case of postoperative peritonsillar, neck phlegmon or neck abscess. No patient reported recurrence of LNC.

**Conclusions:**

Sole complete resection of LNCs is sufficient to avoid postoperative infections and recurrences. Therefore, ipsilateral tonsillectomy and tract dissection is not necessary in routine cases of LNC surgery.

**Supplementary Information:**

The online version contains supplementary material available at 10.1007/s00405-022-07542-0.

## Introduction

The debate on the etiology of lateral neck cysts (LNC) goes back decades. To this date there is no clear evidence for one mechanism of their development. This is reflected in the number of terms being used to describe them, including “branchial cleft cysts”, “congenital neck cysts” or “lymphoepithelial cysts. According to the current doctrine, two major theories exist. The “Branchial Theory” is based on the historical impression of Ascherson (1832) that lateral cervical cysts developed at the site occupied by the branchial apparatus in the embryo. Thus, LNCs are thought to represent a remnant of the structures lying in the lateral aspect of the embryo (pharyngeal cleft/pouch, cervical sinus, thymopharyngeal duct) [1, 2]. The second theory suggests that LNCs originate from cystic transformation of cervical lymph nodes containing squamous epithelium from the tonsil, proposing the name lateral lymphoepithelial cyst [3]. The squamous epithelium in the lymph nodes either represents an embryonic malformation or is washed out of the tonsils into the cervical lymph nodes during infections [4]. Support for the association between cervical cysts and lymphoid tissue is provided by the fact that they mainly occur during adulthood and have a close correlation of their occurrence to inflammation of the throat. In addition, inflammation of LNCs occur as easy as inflammation of lymph nodes. On the other hand, the similarities between tonsil tissue and LNCs could also be clarified by the fact that both derive from the second pharyngeal pouch and thus the attachment of a lateral cervical cyst would already be present at birth [5].

Despite the origin of LNCs, the recommended surgical therapy is inconsistent. In addition to a complete removal of the cyst, which is not questioned by any author, the simultaneous search for a fistulous or obliterated tract to the pharynx and removal if present as well as ipsilateral tonsillectomy is, however, recommended “where applicable” by some authors [5–7]. Whether the reason for tonsillectomy is to remove the origin of this tract, to remove the inflammatory focus or to prevent the re-wash out of squamous epithelium, is not clearly described in literature. Neither does literature answer the question if LNC resection without searching and removing a fistulous tract or its remnants and without tonsillectomy leads to more postoperative recurrences, wound infections or cervical abscesses in this area. In fact, tonsillectomy is performed regularly as part of a LNC operation. Literature on surgical strategies state that the duct of the cyst, which may be difficult to be differentiated from fascial attachments, shall be traced to its origin in the aerodigestive system [8, 9].

The aim of this study was to investigate if tonsillectomy and tract dissection should be performed during regular LNC surgeries and whether the patients suffer from postoperative complications or recurrences without these surgical steps.

## Materials and methods

Prior to this retrospective study approval of the Ethics committee of the University of Regensburg (Registration-No. 21-2214-101) was obtained.

Initially we reviewed the data of all patients who underwent surgery with the ICD-10 diagnostic code Q18.0 (branchiogenic sinus, fistula and cyst) at the Ear Nose and Throat Department of the University Hospital Regensburg between 2004 and 2017. During this period of time, tonsillectomy and tract dissection was generally not performed at our clinic as part of LNC surgery, since it was assumed that LNCs are of lymphoid origin. Patients` data was collected from the clinic's patient management system. Thereof we extracted all those patients who received surgery for LNC without tonsillectomy and without dissection of any fistulous tract. We reviewed clinical diagnosis, operation report and pathological results.

For the analysis of postoperative events or complications, the first step was to screen the medical records of all patients for follow-up visits or admissions related to complications of LNC resection including wound infections, pharyngitis, tonsillitis, peritonsillar abscess or neck infections, such as cervical phlegmon or cervical abscesses. To avoid losing patients during follow-up, who had complications or recurrences treated in other institutions, we planned to carry out a telephone visit of all selected patients.

Therefore, the identified patients were sent a letter informing them about the study. One month later they were contacted by telephone by one of three interviewing physicians, all being consultants for otorhinolaryngology. The interview (see Appendix 1) started with a presentation of the interviewing physician and a verification of the identity of the patient based on his personal data (name/date of birth/date of operation), then the study was briefly described. After informed consent was obtained, the participants were free to arrange a new appointment for a telephone interview or to start the survey immediately. Next step was a structured telephone interview to obtain information on the occurrence of pharyngitis, tonsillitis and neck infections in advance and after the operation, respectively. We also asked about recurrence of LNC. The full questionnaire is described in Appendix 1. Finally, only patients who completed the telephone survey were included in data analysis.

## Results

Inclusion criteria were met by 193 patients. According to our medical records none of these patients represented or were readmitted for tonsillitis, peritonsillar abscess, cervical abscess, cervical phlegmon or recurrent LNC to our clinic. One patient developed a postoperative wound infection before he was discharged after surgery. Reopening, irrigation of the wound and antibiotic treatment was necessary. After 5 years, the patient was seen again in our clinic because of a different clinical picture (midfacial fracture). Until then, no further infection referred to LNC or recurrence of LNC had occurred. This patient had already undergone tonsillectomy prior to LNC surgery. One patient presented again during follow-up due to impairment of wound healing. A comparison of patients characteristics of the two groups are displayed in Table 1. The baseline characteristics of both groups do not show relevant differences.

**Table 1:** Comparison of patients who were included in the study and those who were not available for interview

	Value	Patients included in the study	Patients not available by phone
Number of patients	Total (%)	126 (65)	67 (35)
Age at time of operation	Mean (± SD)	38 (± 14.6)	33(± 14.2)
Gender (female/male)	Total (%)	66/60 (52/48)	32/35 (48/52)
Tonsillectomy before LNC surgery	Total (%)	29 (23)	13 (19)
No Tonsillectomy before LNC surgery	Total (%)	97(77)	54 (81)
Median follow-up time	Total (range)	7 (3–18)	10 (4–15)
Peritonsillar abscess at UKR follow-up	Total (%)	0 (0)	0 (0)
Tonsillectomy at UKR during follow-up	Total (%)	1 (0.8)	0 (0)
Cervical abscess at UKR during follow-up	Total (%)	0 (0)	0 (0)
Cervical infection/phlegmon at UKR during follow-up	Total (%)	1 (0.8)	0 (0)
Wound infection at UKR after LNC surgery	Total (%)	2 (1.6)	2 (3)
Recurrence at UKR of lateral neck cyst	Total (%)	0 (0)	0 (0)

All informations about the group of patients who were not available by phone only refer to the documentation of the Ear Nose and Throat Department of the University Hospital Regensburg (UKR)

**Fig. 1:**
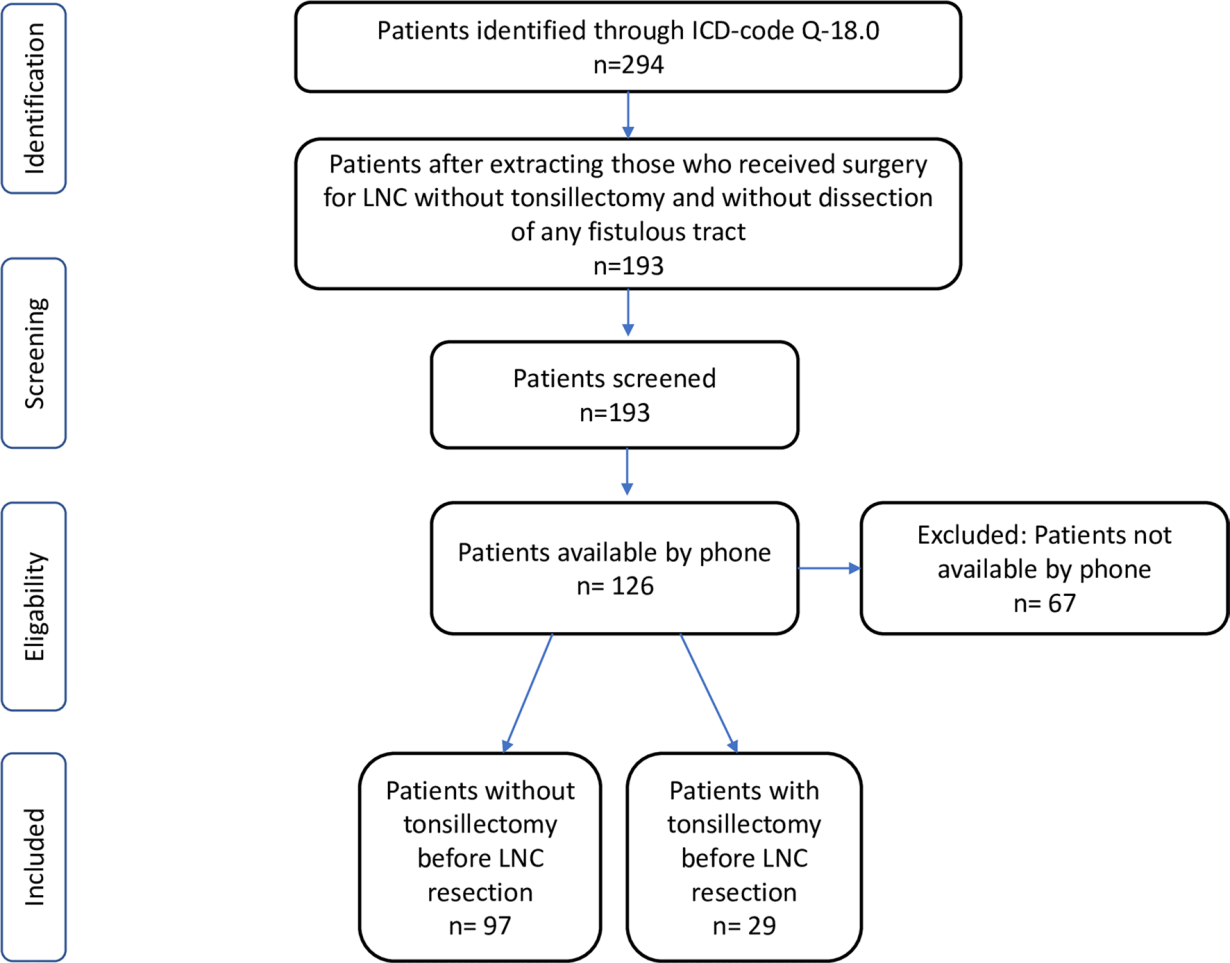
Flow diagram of the patient selection process

In the telephone survey we were able to reach and interview 126 patients (66 female/60 male). All patients whom we were able to reach by telephone and thus inform about the study participated in the study. A flow diagram of the patient selection process is shown in Fig. 1. The results are displayed in Table 2. Mean age at time of operation was 38 years (± 14.6), ranging from 11 to 72. The median follow-up time was 7 years (range 3–18 years/mean follow-up time 11 years). Twenty-nine patients (23%) had undergone tonsillectomy in advance, whereas 97 (77%) patients still had their tonsils in situ at time of the LNC resection. One patient (0.8%) had tonsillectomy performed after the resection of the LNC. This patient developed contralateral LNC during follow-up and had bilateral tonsillectomy performed in the course of its’ resection at another hospital. This was the only patient to indicate that he developed a second LNC on the contralateral side (Fig. 1).

**Table 2:** Incidence of infections of tonsils, pharynx and ipsilateral neck before and after lateral neck cyst surgery

	Yes total (%)	No total (%)
Recurrent tonsillitis and/or pharyngitis before the operation	19 (15)	107 (85)
Recurrent tonsillitis and/or pharyngitis exclusively during childhood	30 (23.8)	96 (76.2)
Cervical infection/phlegmon/abscess before the operation	10 (7.9)	116 (92.1)
Recurrent tonsillitis and/or pharyngitis during follow-up	8 (6.3)	118 (93.7)
Unilateral infections of the pharynx/tonsil during follow-up	0 (0)	126 (100)
Peritonsillar abscess during follow-up	0 (0)	126 (100)
Tonsillectomy during follow-up	1 (0.8)	125 (99.2)
Cervical abscess during follow-up	0 (0)	126 (100)
Cervical infection/phlegmon during follow-up	1 (0.8)	125 (99.2)
Wound infection after LNC surgery	2 (1.6)	124 (98.4)
Recurrence of lateral neck cyst	0 (0)	126 (100)

When asked about tonsillitis or pharyngitis before the operation of the LNC, 30 patients (23.8%) stated that they have had recurrent tonsillitis and/or pharyngitis in childhood. Nineteen patients (15%) answered that they experience recurrent tonsillitis and/or pharyngitis in general. Furthermore, eight patients (6.3%) responded that they had recurrent tonsillitis or pharyngitis after LNC resection, whereof two patients (1.6%) had not reported tonsillitis or pharyngitis before surgery of the LNC. Taking a closer look at the survey of these two patients, one patient described to have pharyngitis twice per year. The second patient stated that he had tonsillitis twice a year for 5 years after LNC surgery which then ceased. Both patients were treated with anti-inflammatory medication without the need for antibiotics. Unilateral infections of the pharynx or tonsil on the side of operation have not been experienced during follow-up by any of the participants. Furthermore, none of the participants experienced peritonsillar abscess or cervical abscess on the side which was operated on during follow-up. Three patients (2.4%) experienced cervical infections after LNC surgery. Thereof two patients (1.6%) had a postoperative wound infection, one of which had intolerance to the suture material. One patient (0.8%) developed a superficial cervical infection at the site of the surgical scar 14 years after the operation. He was treated with oral antibiotics for one week.

## Discussion

Complete surgical resection of LNC is the standard of care. Improper or incomplete resection will result in a high recurrence rate [10]. Furthermore, simultaneous resection of the ipsilateral tonsil and preparation of any fistulous or obliterated tract to the pharynx is frequently recommended [6, 7, 11, 12]. Literature in which the reason for this procedure is justified is rare. Based on the “Branchial Theory” of a common etiology between cysts, sinuses and fistulas, the treatment strategies seem to be put into a nutshell [10, 13, 14]. However, unlike lateral cervical fistulas, which usually present in the first decade, cysts occur in the adolescent and adult [15]. This is also consistent with the results of our study in which the mean age at time of operation was 38 years (± 14.6). According to the medical guiding principle “*primum nihil nocere*, *secundum cavere*, *tertium sanare*” a risk–benefit analysis of these two questionable operation steps (tract preparation/ipsilateral tonsillectomy) is advisable. During the period of data analysis simultaneous tonsillectomy or focused exploration of the neck to dissect any suspected fistulous tract was not performed routinely at our hospital. Lateral neck cysts can be located at different sites along the sternocleidomastoid muscle. According to the congenital theories they belong to the branchial cleft anomalies, of which the second branchial cleft cysts make up approximately 95% [13]. They are categorized according to Bailey (types I–IV) and may have a close relationship to the lower part of the facial nerve, the hypoglossal nerve, the internal jugular vein and the internal and external carotid arteries. In general, injury of neurovascular structures may occur during dissection from the cystic wall to the pharynx exploring the cervical and parapharyngeal region for a tract [16]. Waldhausen stated that local exploration for a fistulous tract shall be performed during LNC surgery and care must be taken to completely excise the tract to prevent recurrences [13]. Advocates of the “Lymph Node Theory” suggest that LNCs on the one hand and fistulas and sinuses on the other hand are of different origin. They recommend that further exploration of the neck to encounter fistulous structures shall not be performed as it will always be unsuccessful in the end [17, 18]. In a recent study on the etiology of branchial cleft anomalies, none of the 42 patients with a lateral neck cyst was found to have a fistulous opening into the pharynx [19].

Tonsillectomy is one of the most common procedures in otorhinolaryngology. Postoperative hemorrhage is the most severe complication and may result in readmission, further surgery and requirement of blood transfusion to control bleeding. It even can be fatal in rare cases. The bleeding rates ranges depending on its classification. In investigations with a large number of participants such as Windfuhr et al. (7132 patients) and Sarny et al. (4594 patients) postoperative hemorrhages requiring intervention ranged from 2.86 to 4.9% [20, 21]. Other complications from tonsillectomy include trauma to adjacent structures, such as pharyngeal wall or teeth and throat pain [22]. To the best of our knowledge a study on whether performing an ipsilateral tonsillectomy is beneficial during resection of lateral neck cyst has not been published. Cheng et al. have questioned this procedure when a true fistula into the pharynx exists. They found no statistically significant difference for recurrence between the group of patients that underwent tonsillectomy (14 patients) and those that did not (22 patients). Limitation of this study was a missing scheduled surveillance after one short-term follow-up visit and the small sample size. In our retrospective series with a median follow-up time of 7 years, we included 126 patients who underwent surgical resection of LNC without tonsillectomy and neck exploration of a fistulous tract. None of our patients developed postoperative infections with symptoms of unilateral pharyngitis, tonsillitis, peritonsillar abscess, neck phlegmon or neck abscess. This was also consistent with the subgroup of patients who had not received tonsillectomy prior to LNC resection (n=97/ 77%). The rate of recurrence of LNC in our series was 0%. The presented results of the study allow the authors to conclude that in routine cases of lateral neck cyst surgery, complete resection without ipsilateral tonsillectomy and tract exploration/resection is sufficient. Due to the possibility that external fistula tracts and orifices may not be visible preoperatively, informed consent should always include excision of fistulous tracts and tonsillectomy to avoid two-stage surgery. The strength of this study is the long follow-up period and the relatively large number of participating patients. There are, however, certain limitations of the study that have to be considered. These include the retrospective nature of the study and a missing standardized clinical re-examination of the patients to sort out inapparent recurrent LNCs with ultrasound. However, a prospective study may not be practicable and reasonable as on the one hand the rate of events is too low and on the other hand the population to treat to high, respectively.

## Conclusions

In our retrospective study, none of the 126 patients who received complete LNC resection only, did experience infections that could be related to remnants of a fistulous tract. Furthermore, within 7 years of median follow-up time (range 3–18 years) we did not see any recurrence. In conclusion, these results do not support the surgical sub-steps of ipsilateral tonsillectomy and exploration for a fistulous or obliterated tract to the pharynx in regular cases of LNC resection.

## Supplementary Information

Below is the link to the electronic supplementary material.

Supplementary file1 (DOCX 20 KB)

## References

[1] Golledge J, Ellis H (1994). The aetiology of lateral cervical (branchial) cysts: past and present theories. J Laryngol Otol.

[2] Janfaza P (2001). Surgical anatomy of the head and neck.

[3] King ES (1949). The lateral lympho-epithelial cyst of the neck; branchial cyst. Austral N Z J Surg.

[4] Stoll W, Hüttenbrink KB (1982). The lateral cervical cyst as a cystic lesion of a lymph node (author’s transl). Laryngol Rhinol Otol.

[5] Hosemann W, Wigand ME (1988). Are lateral neck cysts true derivatives of cervical lymph nodes?. HNO.

[6] Zenner HP (1997). Lehrbuch hals-nasen-ohren-heilkunde.

[7] Guntinas-Lichius O, Klußmann J, Lang S (2021). Referenz HNO-heilkunde.

[8] Houck J (2005). Excision of branchial cysts. Oper Tech Otolayngol Head Neck Surg.

[9] Bailey BJ, Johnson JT, Kohut RI (1993). Head and neck surgery—otolaryngology.

[10] Zaifullah S, Yunus MRM, See GB (2013). Diagnosis and treatment of branchial cleft anomalies in UKMMC: a 10-year retrospective study. Eur Arch Otorhinolaryngol.

[11] Naumann HH, Helms J, Herberhold C, Kastenbauer E (1995). Oto-rhino-laryngologie in klinik & praxis.

[12] Waldhausen JHT (2006). Branchial cleft and arch anomalies in children. Semin Pediatr Surg.

[13] LaRiviere CA, Waldhausen JHT (2012). Congenital cervical cysts, sinuses, and fistulae in pediatric surgery. Surg Clin N Am.

[14] Adams A, Mankad K, Offiah C, Childs L (2016). Branchial cleft anomalies: a pictorial review of embryological development and spectrum of imaging findings. Insights Imaging.

[15] Telander RL, Deane SA (1977). Thyroglossal and branchial cleft cysts and sinuses. Surg Clin N Am.

[16] Al-Mufarrej F, Stoddard D, Bite U (2017). Branchial arch anomalies: Recurrence, malignant degeneration and operative complications. Int J Pediatr Otorhinolaryngol.

[17] Ganz H, Schätzle W, Chilla R (1994). HNO praxis heute.

[18] Bhaskar SN, Bernier JL (1959). Histogenesis of branchial cysts: a report of 468 cases. Am J Pathol.

[19] Pupić-Bakrač J, Skitarelić N, Pupić-Bakrač A (2021). Branchial cleft anomalies: hybrid “branchial inclusion” theory. Eur Arch Otorhinolaryngol.

[20] Windfuhr JP, Chen YS, Remmert S (2005). Hemorrhage following tonsillectomy and adenoidectomy in 15,218 patients. Otolaryngol Head Neck Surg.

[21] Sarny S, Habermann W, Ossimitz G, Stammberger H (2012). The Austrian Tonsil Study 2010—part 2: postoperative haemorrhage. Laryngorhinootologie.

[22] Johnson LB, Elluru RG, Myer CM (2002). Complications of adenotonsillectomy. Laryngoscope.

