# Supplementary material for: Lateral neck cyst surgery without ipsilateral tonsillectomy: a retrospective analysis
Source: Eur Arch Otorhinolaryngol. 2022 Jul 19;280(1):315–20. doi: 10.1007/s00405-022-07542-0 (PMC9813102; doi:10.1007/s00405-022-07542-0)
Supplement: Supplementary file 1 — Supplementary file1 (DOCX 20 KB) [file 405_2022_7542_MOESM1_ESM.docx]

**Appendix:**

**Lateral neck cyst surgery without ipsilateral tonsillectomy –**

**a retrospective analysis**

**for**

**European Archives of Otorhinolaryngology**

Franziska Schwan, M.D.^1^, Julian Künzel, M.D.^1^, Florian Weber, M.D.^2^, Veronika Vielsmeier, M.D.^1^, Christopher Bohr, M.D.^1^, Kornelia E. C. Andorfer, M.D. (ORCID: **0000-0002-5195-0634)** ^1^

^1^ Department of Otorhinolaryngology, University Hospital Regensburg, Franz-Josef-Strauss-Allee 11, Regensburg D-93053, Germany

^2^ Department of Pathology, University Hospital Regensburg, Franz-Josef-Strauss-Allee 11, Regensburg D-93053, Germany

Address correspondence to Kornelia E. C. Andorfer, [Kornelia.Andorfer@ukr.de](mailto:Kornelia.Andorfer@ukr.de)

**Telephone interview**

**Part 1**

Date:______________

1. Introduction of Interviewer
2. Name of patient/ date of birth:
3. Have you received our information leaflet? ⃝ yes ⃝ no
4. brief description of the study
5. Information on the voluntary nature of participation in the study
6. Consent to participate in the study
7. Arrangement of a second telephone appointment/ if necessary date

**Part 2**

1. You have received the operative resection of a lateral neck cyst ……………(date)

on the right ⃝ left ⃝ side of the neck.

1. Did you have tonsillectomy before this operation?

yes ⃝ no ⃝ If yes,

why_______________________________________

1. A) Did you have recurrent tonsillitis and/or pharyngitis before the operation?

yes ⃝ no ⃝

how often (e.g. twice/year)?_______________________________

type of therapy (e.g. antibiotics)?___________________________________

B) Did you have recurrent tonsillitis and/or pharyngitis only in childhood?

yes ⃝ no ⃝

how often? __________________________________________

type of therapy?__________________________________________

1. Did you have (recurrent) infections of the neck (ipsilateral) before the operation?

yes ⃝ no ⃝

what kind (phlegmon/ abscess/ infected LNC)?___________________

how often?__________________________________________

type of therapy?__________________________________________

1. Did you have recurrent tonsillitis and/or pharyngitis or monolateral tonsillitis/ pharyngitis (ipsilateral) after the operation?

yes ⃝ no ⃝ if yes, description__________________________

how often?__________________________________________

type of therapy?__________________________________________

1. Did you have peritonsillar abscess after the operation?

yes ⃝ no ⃝

If yes, which kind of treatment?

Draining of abscess ⃝

Ipsilateral tonsillectomy ⃝

Antibiotic treatment ⃝

(approximate) date ________________________

1. Did you have tonsillectomy in the interim (during follow-up)?

yes ⃝ no ⃝

If yes, state reason.

Infections ⃝

snoring ⃝

tumor ⃝

Others :­­­­­­­­­­­­­­­­­­­­­­­­­­­­­_________________________________

1. Did you have a cervical abscess (ipsilateral) after the operation?

yes ⃝ no ⃝

If yes, which treatment?

Operative drainage ⃝

antibiotics ⃝

1. Did you have a cervical infection or wound healing disorder after the operation?

yes ⃝ no ⃝

Further explain______________________________________________

If yes, which treatment?

Operative drainage ⃝

antibiotics ⃝
